# Brain-derived neurotrophic factor plasma levels are associated with mortality in critically ill patients even in the absence of brain injury

**DOI:** 10.1186/cc11902

**Published:** 2012-12-17

**Authors:** Cristiane Ritter, Aline S Miranda, Vinícius Renê Giombelli, Cristiane D Tomasi, Clarissa M Comim, Antonio Lucio Teixeira, João Quevedo, Felipe Dal-Pizzol

**Affiliations:** 1 Laboratório de Fisiopatologia Experimental and Instituto Nacional de Ciência e Tecnologia Translacional em Medicina, Programa de Pós-Graduação em Ciências da Saúde, Unidade Acadêmica de Ciências da Saúde, Universidade do Extremo Sul Catarinense, Av. Universitária, 1105, Criciúma, 88806000, Brazil; 2 Intensive Care Unit, Hospital São José, Cel. Pedro Benedet, 630, Criciúma, 88801250, Brazil; 3 Grupo de Neuroimunologia, Laboratório de Imunofarmacologia, Departamento de Bioquímica e Imunologia, Instituto de Ciências Biológicas, Universidade Federal de Minas Gerais, Av. Antonio Carlos, 6627, Belo Horizonte, 31270901, Brazil; 4 Laboratório de Neurociências and Instituto Nacional de Ciência e Tecnologia Translacional em Medicina, Programa de Pós-Graduação em Ciências da Saúde, Unidade Acadêmica de Ciências da Saúde, Universidade do Extremo Sul Catarinense, Av. Universitária, 1105, Criciúma, 88806000, Brazil

## Abstract

**Introduction:**

Because of its relevance to the functioning of the central nervous system, brain-derived neurotrophic factor (BDNF) has been implicated in the pathogenesis of different neuropsychiatric diseases. Whether the BDNF level can be a marker of brain dysfunction and thus predict mortality in critically ill patients is not known. Thus we aimed to determine whether the plasma levels of BDNF are associated with morbidity and mortality in critically ill patients.

**Methods:**

Healthy volunteers (*n *= 40) and consecutive patients older than 18 years (*n *= 76) admitted for more than 24 hours in an Intensive Care Unit (ICU) in a University hospital between July and October 2010 were included in the present study. First blood samples were collected within 12 hours of enrollment (D0), and a second sample, 48 hours after (D2) for determination of plasma BDNF levels. The relation between BDNF levels and mortality was the primary outcome. The secondary outcomes were the relation between BDNF levels and delirium and coma-free days (DCFD) and ICU and hospital length of stay (LOS).

**Results:**

Admission plasma levels of BDNF were higher in ICU patients when compared with healthy volunteers (1,536 (962) versus 6,565 (2,838) pg/ml). The mean BDNF D2 was significantly lower in nonsurvivor patients (5,865 (2,662) versus 6,741 (2,356) pg/ml). After adjusting for covariates, BDNF levels, the need for mechanical ventilation, and sepsis were associated with mortality. Even in patients without clinically detectable brain dysfunction, lower BDNF D2 levels were associated with mortality. BDNF D2 had a mild correlation to DCFD (*r *= 0.44), but not to ICU and hospital LOS. In addition, plasma BDNF did not correlate to different plasma cytokines and platelets levels.

**Conclusions:**

The plasma levels of BDNF were independently associated with mortality, even in the absence of clinically detectable brain dysfunction.

## Introduction

Neurotrophins are considered to play a major role in neural function including survival, development, and plasticity. Brain-derived neurotrophic factor (BDNF) is the most abundant neurotrophin in the mammalian central nervous system (CNS). BDNF protects the CNS from injury, at least in part, by inhibiting apoptosis [1] and by stimulating sprouting and neuronal reorganization [2]. Because of its relevance to the functioning of the CNS, BDNF has been implicated in the pathogenesis of different neuropsychiatric diseases, including depression, brain trauma, and schizophrenia [3-5]. Recently, BDNF was implicated in metabolic regulation [6], in the development of lung diseases [7], and in the support of hematopoiesis [8].

Brain dysfunction is a frequent complication of the critically ill patients. It is manifested mainly as coma or delirium and has a great impact on morbidity and mortality of critically ill patients. Despite its importance, delirium is frequently underdiagnosed, even when systematically assessed with validated tools [9]. Thus, the search for biomarkers of brain dysfunction is an important field in critical care research.

An increase in the levels of S100β (a calcium-binding protein expressed in the cytoplasm of astroglia and Schwann cells) had a positive correlation with lactate levels and negatively with mean arterial pressure in critically ill patients without evident brain injury [10]. In addition, neuronal specific enolase (NSE) and S100β did not correlate to outcome in critically ill patients [11]. We demonstrated, in a case-control study, that BDNF was associated with delirium occurrence, but not with mortality, in this subgroup of patients [12]. If BDNF levels can be a marker of subclinical brain dysfunction and thus predict morbidity and mortality in critically ill patients is not known. We hypothesized that, even in the absence of clinically detectable brain dysfunction, plasma levels of BDNF are associated with morbidity and mortality in critically ill patients.

## Materials and methods

### Patients

A prospective cohort study was conducted in a convenience sample of patients older than 18 years admitted for more than 24 hours to an ICU in a University hospital between July and October 2010. Patients were excluded if they had persistent coma or if they were admitted as a result of brain trauma, postcardiac arrest, or other neurologic reason (for example, stroke). Forty healthy volunteers paired for sex and age were included, and fasting blood was collected to determine the plasma levels of BDNF. Hospital Sao José and Universidade do Extremo Sul Catarinense review boards approved this study. Informed consent was obtained from all patients or their relatives before study inclusion.

### Procedures

Demographic and clinical variables were collected and allowed us to determine the Acute Physiology and Chronic Health Evaluation (APACHE) II score [13] and the Sequential Organ Failure Assessment (SOFA) score [14]. Severe sepsis and septic shock were defined according to internationally agreed-on criteria [15]. Patients were screened for delirium twice a day until ICU discharge by using the Confusion Assessment Method for the Intensive Care Unit (CAM-ICU) [16], by trained researchers. Patients were diagnosed with delirium when they had at least one positive CAM-ICU screening. Coma was defined as a Richmond Agitation Sedation Scale (RASS) score of -4 or -5. Clinically detectable brain dysfunction was defined as the presence of either delirium or coma. The first blood sample was collected within 12 hours of ICU admission (D0), and a second one, 48 hours after (D2). Blood was collected in Vacutainers with citrate and immediately centrifuged at 3,000 *g *for 10 minutes, at 4°C. The plasma was collected and stored at -80°C. Plasma levels of BDNF and soluble tumor necrosis factor receptors (STNFR) 1 and 2, interleukin (IL)-1β, IL-6, and IL-10 were measured with enzyme-linked immunosorbent assay (ELISA), according to the procedures supplied by the manufacturer (DuoSet; R&D Systems, Minneapolis, MN, USA). All samples were assayed in duplicate. Lower detection limits for BDNF, sTNFRs, IL-1, IL-6, and IL-10 were 5, 10, 3, 3, and 6 pg/ml, respectively. Concentration was expressed as pictograms per milliliter.

The relation between BDNF levels and mortality was the primary outcome. As secondary outcome, we chose the relation between BDNF levels and DCFD (that indicate the days during the study period that a patient was alive and free of acute brain dysfunction), ICU, and hospital length of stay (LOS). Once BDNF could be related to the inflammatory response and released from platelets, the correlation between plasma levels of BDNF and different inflammatory molecules, as well platelets levels, was determined.

### Statistical analysis

All variables were tested for normality by using the Kolmogorov-Smirnov test. Differences in baseline characteristics and BDNF levels between survivor and nonsurvivor patients and between healthy volunteers and ICU patients were tested by using χ^2 ^tests and the *t *test or Mann-Whitney test. The area under receiver operating characteristic (AUROC) curve was used to evaluate the ability of BDNF to predict mortality.

The percentage variation of BDNF was calculated by using the formula ((BDNF D2 - BNDF D0)/BDNF D0 × 100) and dichotomized into "decrease" or "no decrease." Decrease was considered a reduction compared with D0 levels higher than 25%. To examine the association between BDNF and mortality, a binary logistic regression was performed. The model was adjusted for physiological changes that would confound the relation between BDNF and survival. Thus disease severity, age, need for mechanical ventilation, sepsis, need for vasoactive drugs, and need for sedation were incorporated into the model. The need for vasoactive drugs is already incorporated in the SOFA score. Thus, to avoid double-counting, it was not used as a separate covariate in the model. Co-linearity was found between the use of sedation and mechanical ventilation. Thus the model used the SOFA score (and not the APACHE score, as a variable associated with disease severity), age, need for mechanical ventilation, sepsis, and BDNF levels. BDNF entered into the model as the odds among patients at the 25^th ^percentile of BDNF levels versus all other quartiles grouped (a more clinically relevant approach: Model 1) or as the generally used 1-unit change in its D2 levels (Model 2).

The correlation between BDNF and DCFD, ICU, or hospital LOS was determined by the Pearson test. A multivariate linear regression was performed to determine the association between plasma BDNF and DCFD, and it included the same parameters described for the binary regression. The correlation between plasma BDNF and plasma cytokines or platelet levels was performed with the Pearson test. A sample size of 17 patients in each group was necessary to detect a 50% difference in biomarkers between groups. Statistical significance was defined as a *P *value < 0.05.

## Results

In total, 76 patients were included, 56 survivor and 20 nonsurvivor. The differences between survivors and nonsurvivors are presented in Table 1. The main admission reasons were cardiovascular (*n *= 9), respiratory failure (*n *= 13), sepsis (*n *= 25), and postoperative monitoring (*n *= 17), with no statistical significant differences between survivors and nonsurvivors (*P *= 0.321). Only four patients received immunosuppressants, and nine patients were given renal replacement therapy, with no statistical significant differences between survivors and nonsurvivors (*P *= 0.674 and *P *= 0.611, respectively).

**Table 1:** Characteristics of patients enrolled in the present study

	All patients (*n *= 76)	Survivors (*n *= 56)	Nonsurvivors (*n *= 20)	*P *value
Age, years, mean (SD)	55 (19)	52 (19)	62 (16)	0.04
Sex, male, *n *	52	40	12	0.345
Admission, *n*				
Surgical	27	20	7	0.954
Clinical	49	36	13	
Origin, *n*				
Ward	34	22	12	0.181
Emergency room	42	34	8	
Sepsis, during ICU stay, *n*	39	23	16	0.003
APACHE II score, points, mean (SD)	19 (8)	18 (8)	20 (8)	0.319
SOFA admission, points, mean (SD)	6.6 (4.0)	6.0 (3.4)	8.3 (4.7)	0.03
Mechanical ventilation during ICU stay, *n*	46	29	17	0.008
Sedation during ICU stay, *n*	50	32	18	0.008
Vasopressor need during ICU stay, *n*	31	21	10	0.329
BDNF D0 mean (SD)	4,831 (3,366)	6,861 (2,764)	5,736 (2,951)	0.129
BDNF D2, mean (SD)	5,865 (2,661)	6,741 (2,356)	3,413 (1,813)	< 0.0001
% variation BDNF, *n *				
Decrease	19	8	11	0.001
No change/increase	57	48	9	

APACHE II, Acute Physiology and Chronic Health Evaluation score; BDNF D0, brain-derived neurotrophic factor collected within 12 hours of enrollment; BDNF D2, brain-derived neurotrophic factor collected 48 hours after intensive care admission; ICU, intensive care unit; SD, standard deviation; sepsis, severe sepsis and septic shock; SOFA, sequential organ-failure assessment.

BDNF D0 levels were higher when compared with those of healthy volunteers (1,536 ± 962 versus 6,565 ± 2,838 pg/ml; *P *< 0.0001), but were not significantly different when compared between survivors and nonsurvivors (Table 1). In contrast, BDNF D2 levels were significantly lower in nonsurvivor patients (Table 1), with an AUROC curve to predict survival of 0.87 (0.78 to 0.95). This was similar when BDNF levels were transformed as a percentage variation (Table 1). After adjusting for covariates, BDNF and the need for mechanical ventilation or sepsis were associated with mortality (Table 2).

**Table 2:** Multivariate analysis of characteristics associated with mortality

	OR (CI 95%)	*P *value
Model 1		
BDNF 25^th ^quartile	12.7 (2.2 to 73)	0.004
Sepsis	6.3 (1.1 to 39)	0.04
Age	1.01 (0.98 to 1.06)	0.33
Admission SOFA	1.06 (0.87 to 1.31)	0.53
Mechanical ventilation	8.56 (0.92 to 79)	0.05

Model 2		
BDNF D2	29 (4 to 200)	0.001
Sepsis	3.3 (0.56 to 20)	0.18
Age	1.04 (0.99 to 1.09)	0.09
Admission SOFA	0.91 (0.72 to 1.15)	0.46
Mechanical ventilation	14 (1.1 to 175)	0.038

Hosmer and Lemershow goodness-of-fit model 1, χ^2 ^= 2.2; *P *= 0.97; and model 2, χ^2 ^= 1.9; *P *= 0.98. BDNF D2, brain-derived neurotrophic factor collected 48 hours after intensive care admission; CI, confidence interval; OR, odds ratio; SOFA, sequential organ-failure assessment.

To ascertain further that the presence of clinically detectable brain dysfunction did not interfere in these results, as an association between BDNF and mortality could simply represent clinically detectable brain dysfunction, we analyzed the ability of BDNF to predict outcome in patients without brain dysfunction. Even in these patients, the levels of BDNF were associated with mortality (Table 3), as well in the subgroup of patients with clinically detectable brain dysfunction (Table 4).

**Table 3:** Brain-derived neurotrophic factor levels in the subset of patients without clinically detectable brain dysfunction

	Survivors (*n *= 31)	Nonsurvivors (*n *= 8)	*P *value
BDNF D0, mean (SD)	6,811 (2,613)	5,970 (3,054)	0.437
BDNF D2, mean (SD)	6,736 (2,613)	3,115 (1,997)	< 0.0001
% variation BDNF, *n *			
Decrease	4	5	0.003
No change/increase	27	3	

BDNF D0, brain-derived neurotrophic factor collected within 12 hours of enrollment; BDNF D2, brain-derived neurotrophic factor collected 48 hours after intensive care admission.

**Table 4:** Brain-derived neurotrophic factor levels in the subset of patients with clinically detectable brain dysfunction

	Survivors (*n *= 25)	Nonsurvivors (*n *= 12)	*P *value
BDNF D0, mean (SD)	6,923 (2,994)	5,580 (3,006)	0.210
BDNF D2, mean (SD)	6,748 (2,594)	3,612 (1,741)	< 0.0001
% variation BDNF, *n *			
Decrease	4	6	0.049
No change/increase	21	6	

BDNF D0, brain-derived neurotrophic factor collected within 12 hours of enrollment; BDNF D2, brain-derived neurotrophic factor collected 48 hours after intensive care admission.

A significant, but weak, correlation between BDNF D0 and DCFD (*r *= 0.31; *P *= 0.01), as well a mild correlation between BDNF D2 and DCFD (*r *= 0.44; *P *< 0.001) were observed In the linear regression BDNF D2 levels (unstandardized B 6.95 (2.3 to 9.7); *P *= 0.002), but not BDFN D0 (unstandardized B 2.05 (-2.4 to 6.5); *P *= 0.36) and sepsis (unstandardized B -3.4 (-6.7 to -0.08); *P *= 0.045) were independently associated with DCFD. Plasma levels of BDNF did not correlate to either ICU or hospital LOS (data not shown). In addition, plasma levels of BDNF did not correlate to any of the measured plasma cytokines or to platelets levels (data not shown).

## Discussion

We demonstrate here that plasma levels of BDNF are related to mortality, independent of the presence of clinically detectable delirium, and to DCFD, but not to ICU and hospital LOS.

BDNF is thought to play a major role in neural function, including survival and plasticity [17]. The majority of the effects of BDNF are mediated by binding with its specific receptor that is highly expressed in the adult brain and is essential in survival of mature neurons [18]. The observation that the decrease in BDNF levels during the ICU stay is related to mortality suggests that monitoring the levels of BDNF during the ICU stay can improve our ability to predict death in these patients. In addition, we demonstrate that critically ill patients presented higher BDNF plasma levels when compared with healthy volunteers. We suppose that this is related to the fact that BDNF is crucial for the recovering of brain after an acute stress [19,20] and the decrease in the levels of BDNF in nonsurvivors suggests the inability of these patients to cope with acute stress [20]. It is possible that, during critical illness, the central nervous system needs higher levels of BDNF to maintain its function, and the inability to maintain adequate levels of BDNF is associated with brain dysfunction (either clinical or subclinical).

Our results do not allow us to determine a direct relation between BDNF levels and mortality. Actually, the late-onset BDNF-knockout mice grow at a normal rate and can survive more than a year, but have several behavioral deficits [21]. Thus, it seems that BDNF is not essential for survival, at least in animals that are not submitted to stress. This is a relevant next step to be made, to understand whether a direct association exists between BDNF and survival or if our results represent only one more marker of severity in ICU patients.

Brain-specific proteins are reliable markers of brain injury, and predict outcome in head injury, stroke, and postcardiac arrest [22-24]. Plasma levels of S100β and NSE can predict early ICU mortality; plasma levels of S100β measured at ICU admission are independent predictors of survival in sepsis patients [25]. In contrast, Weigand *et al. *[26] demonstrated a relation between plasma levels of NSE, but not S100β, and mortality in sepsis patients [26]. In general ICU patients, we could not demonstrate any significant relation between plasma levels of these markers and mortality [11].

Some limitations occur in the use of S100β and NSE as markers of brain injury in general ICU patients. Cell death must occur for them to be released from the CNS, but probably during nonprimary CNS diseases, large amounts of cell death are not found [27]. Indeed, S100β seems to be a marker of hypoperfusion in critically ill patients [10]. We suggest that plasma levels of BDNF can be a better marker of brain dysfunction in critically ill patients, when compared with S100β and NSE, because it is related to the ability of brain to recover from an injury, and it is not just released from dead neurons. Actually, neuronal activity is necessary to transcribe and release BDNF from neurons, and critical illness-associated organ failure can be characterized as a hypometabolic state with decreased mitochondrial activity and adenosine-5'-triphosphate production [28], reinforcing the idea that the decrease in plasma levels of BDNF can be a reflex of brain dysfunction.

Some limitations must be pointed out. First, this is a pilot investigation and may lack statistical power to detect some clinically important associations. Because of the relatively small sample size, only a limited number of covariates could be incorporated into the regression models. Second, production of BDNF occurs in peripheral tissues [29], and because we measured the levels of BDNF in plasma, and not in cerebrospinal fluid, we cannot provide a definitive confirmation of a brain-specific source of BDNF. Despite this, BDNF can cross the blood-brain barrier [30], and evidence indicates that plasma levels of BDNF are related to its CNS levels, at least in animals [31]. The release of BDNF by peripheral organs, mainly the gut epithelia, is not related to a local function of BDNF in these tissues, because, in general, they lack both high- and low-affinity BDNF receptors [29]. Therefore, it has been postulated that peripheral BDNF is taken up by neurons of the central and peripheral nervous system. In addition, the inflammatory response seems to be involved in the release of BDNF in humans [32], but we cannot find any statistically significant correlation between the levels of BDNF and several cytokines. This suggests that the decrease in plasma BDNF levels is not related to the inflammatory response. BDNF can be released from platelets, mainly during the clotting process [33], so serum BDNF levels are higher when compared with plasma BDNF levels [34]. Thus, because platelets might serve as a reservoir for circulating BDNF [35], the plasma levels of BDNF do not reflect platelet levels, and this information could be important to understanding our results.

## Conclusions

The plasma levels of BDNF are independently related to mortality, even in the absence of clinically detectable brain dysfunction. This suggests that the monitoring of plasma levels of BDNF can improve our ability to predict outcome in critically ill patients.

## Key messages

• BDNF is suggested to be a surrogate of brain dysfunction in critically ill patients.

• BDNF plasma levels are independently associated with mortality in critically ill patients.

## Abbreviations

APACHE: Acute Physiology and Chronic Health Evaluation; AUROC: area under receiver operating characteristic; BDNF: brain-derived neurotrophic factor; CAM-ICU: Confusion Assessment Method for the Intensive Care Unit; CNS: central nervous system; DCFD: delirium-coma-free days; ELISA: enzyme-linked immunosorbent assay; ICU: intensive care unit; IL: interleukin; LOS: length of stay; NSE: neuron-specific enolase; RASS: Richmond Agitation Sedation Scale; SOFA: Sequential Organ Failure Assessment; STNFR: soluble tumor necrosis factor receptor.

## Competing interests

The authors declare that they have no competing interests.

## Authors' contributions

CR, ALT, JQ, and FDP made substantial contributions to conception and design of the study and were involved in drafting the manuscript. CDT, CMC, VRG, and ASM made substantial contributions to the acquisition of data, its analysis, and interpretation. All authors read and approved the manuscript for publication.
